# Glucosamine reduces the inhibition of proteoglycan metabolism caused by local anaesthetic solution in human articular cartilage: an in vitro study

**DOI:** 10.1186/s40634-017-0106-4

**Published:** 2017-11-13

**Authors:** Abhinav Gulihar, Shalin Shaunak, Pinelopi Linardatou Novak, Parthiban Vinayakam, Baljinder Dhinsa, Grahame Taylor

**Affiliations:** 1 Consultant Orthopaedic Surgeon, Princess Royal University Hospital, Farnborough, Kent UK; 2 Consultant Orthopaedic Surgeon, Leicester General Hospital, Leicester, UK; 3 Orthopaedic Registrar, St Helier Hospital, Sutton, UK; 4 Queen Elizabeth The Queen Mother Hospital, Margate, Kent UK

## Abstract

**Background:**

We assessed whether local anaesthetics caused inhibition of proteoglycan metabolism in human articular cartilage and whether the addition of Glucosamine sulphate could prevent or allow recovery from this adverse effect on articular cartilage metabolism.

**Methods:**

Cartilage explants obtained from 13 femoral heads from fracture neck of femur patients (average age 80 years, 10 female) were exposed to either 1% Lidocaine, 2% Lidocaine, 0.25% Bupivacaine, 0.5% Bupivacaine, 0.5% Levo-bupivacaine or a control solution (M199 culture medium). Glucosamine-6-Sulphate was added during or 1 h after exposure to 0.5% Bupivacaine to assess its protective and reparative effects. After exposure, the explants were incubated in culture medium containing radio labelled 35-sulphate and uptake was measured after 16 h to give an assessment of proteoglycan metabolism.

**Results:**

The reduction in 35-S uptake compared to control was 65% for 1% Lidocaine (*p* < 0.001), 79% for 2% Lidocaine (*p* < 0.001), 61% for 0.25% Bupivacaine (*p* < 0.001), 85% for 0.5% Bupivacaine (*p* < 0.001) and 77% for 0.5% Levobupivacaine (*p* < 0.001). Glucosamine was able to protect the articular cartilage by reducing the inhibition of proteoglycan metabolism of 0.5% Bupivacaine from 85 to 30% (*p* < 0.001). When added after 0.5% Bupivacaine exposure, Glucosamine allowed some recovery with inhibition of metabolism to 70% (*p* = 0.004).

**Conclusion:**

Our results showed that all local anaesthetic solutions inhibited proteoglycan metabolism in articular cartilage and the addition of Glucosamine was able to reduce the inhibition of metabolism caused by 0.5% Bupivacaine. Intra-articular injection of local anaesthetics requires careful consideration of risks and benefits.

## Background

Chondrolysis is a rare but devastating complication of arthroscopic surgery. Postoperative infusion of local anaesthetic solutions, particularly Bupivacaine, has been implicated as one of the causes (Scheffel et al. 2010; Anderson et al. 2010; Anakwenze et al. 2010; Hansen et al. 2007). Recent in vitro and animal in vivo studies have demonstrated that even a single exposure of Lidocaine, Bupivacaine or Ropivacaine can be harmful to articular cartilage (Chu et al. 2010; Chu et al. 2008; Chu et al. 2006; Karpie and Chu 2007; Piper and Kim 2008). Grishko et al. (2010) suggested that this effect was a result of apoptosis and mitochondrial DNA damage in chondrocytes but Bogatch et al. (2010) have suggested that a chemical incompatibility between the local anaesthetic solution and the synovial fluid could be responsible.

Glucosamine is a normal component of human articular cartilage. Laboratory studies have shown that Glucosamine has a protective, reparative and anti-inflammatory action on chondrocytes and articular cartilage (Chan et al. 2005; Parker 2006; Shikhman et al. 2005; Terry et al. 2007). While studies have shown that Glucosamine can reverse articular cartilage damage experimentally induced by chemicals such as lipopolysaccharide and papain (Fenton et al. 2000; Oegema et al. 2002), there are currently no studies to assess whether Glucosamine can reverse the potentially harmful effects of local anaesthetics.

This in vitro study was carried out to test the hypothesis that commonly used local anaesthetic solutions inhibit proteoglycan metabolism in human articular cartilage, and that the addition of glucosamine can protect against these harmful effects.

## Methods

Ethical approval for this study was obtained from the national research ethics committee and local approval was obtained from the hospital research and development department.

We used the established technique of measuring radio labelled sulphate uptake by chondrocytes to form proteoglycans (Lane and Brighton 1974; Meachim and Collins 1962; Mankin and Lippiello 1969). The uptake of sulphate is proportional to the metabolic activity of the chondrocytes (Collins and McElligott 1960; Gulihar et al. 2013; Bulstra et al. 1994).

Femoral heads were retrieved at the time of surgery (hemiarthroplasty) from 13 patients (average age 80 years, 10 female) who had suffered a fractured neck of femur. Inclusion criteria included patients undergoing hip hemiarthroplasty following an intracapsular fractured neck of femur. Exclusion criteria included those with extracapsular fractures which are normally fixed, those with intracapsular fractures with suspicion of malignancy, where femoral heads were sent for histological analysis, and those patients with dementia who could not consent to participation in the study.

In our unit, all hip fractures are operated upon within 36 h of admission to hospital. The femoral heads were stored immediately after retrieval from the patients at 37 degrees C in M199 culture medium supplemented with 10% foetal calf serum, 250 g/ml L-glutamine, 50 g/ml ascorbic acid, 500 IU/ml penicillin and 500 g/ml streptomycin (Sigma-Aldrich Company Ltd., Dorset, UK). This medium has been used for assessing cartilage metabolism in previous studies (Gulihar et al. 2013; Bulstra et al. 1994). In a tissue culture laboratory, 4 mm articular cartilage explants were harvested from the underlying subchondral bone, placed in 100 l of culture medium and weighed.

The specimens were then exposed to one of 8 different experimental conditions for 1 hour each as outlined in Table 1. Explants exposed only to M199 culture medium were used as control. To assess its protective effect, 10 mg Glucosamine-6-sulphate (100 mg/ml solution) was added along with 0.5% Bupivacaine to the specimens in experimental condition 7. To assess its reparative effect, Glucosamine was added after the specimens had been exposed to 0.5% Bupivacaine for 1 hour (experiment 8). The volume of Glucosamine added was only 0.1 ml and hence the dilution was only 10%.

**Table 1:** Experimental conditions used to test the effect of local anaesthetics and Glucosamine on articular cartilage. Radio-labelled sulphur (35-S) uptake was compared to the control solution (M199)

Experiment Condition	Number of specimens	Test solution exposure (for 1 h)	Recovery Incubation (for 16 h)
1	38	1% Lidocaine (1 ml)	M199 (1 ml) + 35-S (5 mCi)
2	38	2% Lidocaine (1 ml)	M199 (1 ml) + 35-S (5 mCi)
3	38	0.25% Bupivacaine (1 ml)	M199 (1 ml) + 35-S (5 mCi)
4	38	0.5% Bupivacaine (1 ml)	M199 (1 ml) + 35-S (5 mCi)
5	38	0.5% Levo-bupivacaine (1 ml)	M199 (1 ml) + 35-S (5 mCi)
6	38	Control - M199 (1 ml)	M199 (1 ml) + 35-S (5 mCi)
7	26	0.5% Bupivacaine (1 ml) + Glucosamine-6-sulphate (10 mg/100μl)	M199 (1 ml) + 35-S (5 mCi)
8	26	0.5% Bupivacaine (1 ml)	M199 (1 ml) + Glucosamine-6-sulphate (10 mg/100μl) + 35-S (5 mCi)

Depending on available macroscopically intact cartilage, a maximum of 24 explants was obtained from each femoral head. This was performed in batches of eight explants obtained from different areas of the femoral head with area dictated by cartilage availability. We ensured that only explants with full thickness intact cartilage were harvested. One explant from each batch of eight was exposed to one of the solutions. We had lower numbers in the Glucosamine groups because of limited availability of Glucosamine-6-Sulphate from the supplier Perkin Elmer. In-spite of this at least 26 explants were used for each experimental condition, much higher than previous studies (Chu et al. 2008; Chu et al. 2006).

After this, the explants were washed three times with culture medium for 20 min each to remove any residual local anaesthetic. Samples were then incubated in M199 containing 5 mCi radio labelled Sulphur-35 (^35^SO_4_)(Perkin Elmer, Cambridge, UK) for 16 h before being washed three times in phosphate-buffered saline (Sigma-Aldrich Company Ltd., Dorset, UK) for 20 min each.

De Vries et al. (Gulihar et al. 2013) calculated that each cycle of washing removed 95% of unbound radioactive sulphate. To measure the amount of radio-labelled sulphur taken up by the chondrocytes, the cartilage was broken down by protease-K (2.5 IU per ml in 0.05 M TriseHCl, 1 mmol CaCl2, pH 7.9; Sigma-Aldrich Company Ltd., Dorset, UK) for 24 h. McKenzie et al. (Bulstra et al. 1994) showed this method liberated 95% of incorporated radio nucleotide.

The liquid was then drawn off and the specimen centrifuged at 10000 rpm for 1 min to remove debris. Three 100 l aliquots from each sample were removed and added to 1 ml Biofluor scintillation fluid (Perkin Elmer, Cambridge, UK). Average measurement of the 3 aliquots were recorded and divided by the weight of the specimen to give counts per gram (CPG) of cartilage per minute in a liquid scintillation counter.

**Fig. 1:**
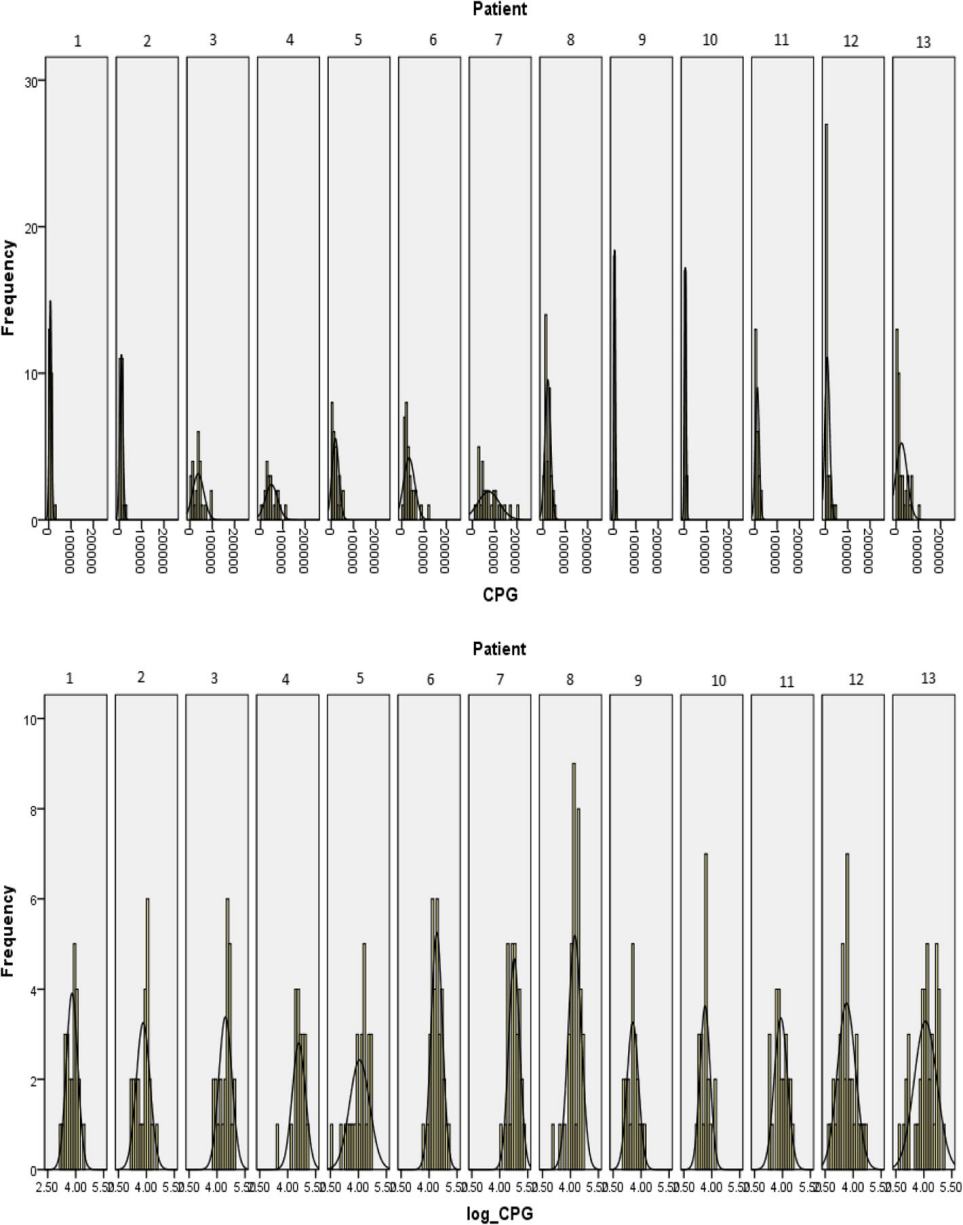
Histograms showing the data distribution of 35-S uptake of each patient before and after log transformation

Descriptive statistics were calculated for the ^35^SO_4_ uptake (CPG) of each local anaesthetic and control. The data distribution was found to be skewed and was transformed by log base 10 to produce a near normal distribution prior to statistical analysis (Fig. 1). Geometric means were calculated for each variable and the percentage difference between the ^35^SO_4_ uptake of control and each variable was calculated as percentage inhibition of metabolism using the equation:

Statistical comparisons were conducted between the ^35^SO_4_ uptake of cartilage specimens exposed to different local anaesthetics and that of the control group, separately for each anaesthetic, using a statistical mixed model taking into account the random effect of each patient. To assess the protective and reparative effect of Glucosamine, a comparison was made between specimens exposed to 0.5% Bupivacaine only with those exposed to 0.5% Bupivacaine and Glucosamine.

Statistical software STATA (StataCorp LP, Texas, USA) was used for the analysis and significance was assumed at *p* < 0.05.

## Results

**Fig. 2:**
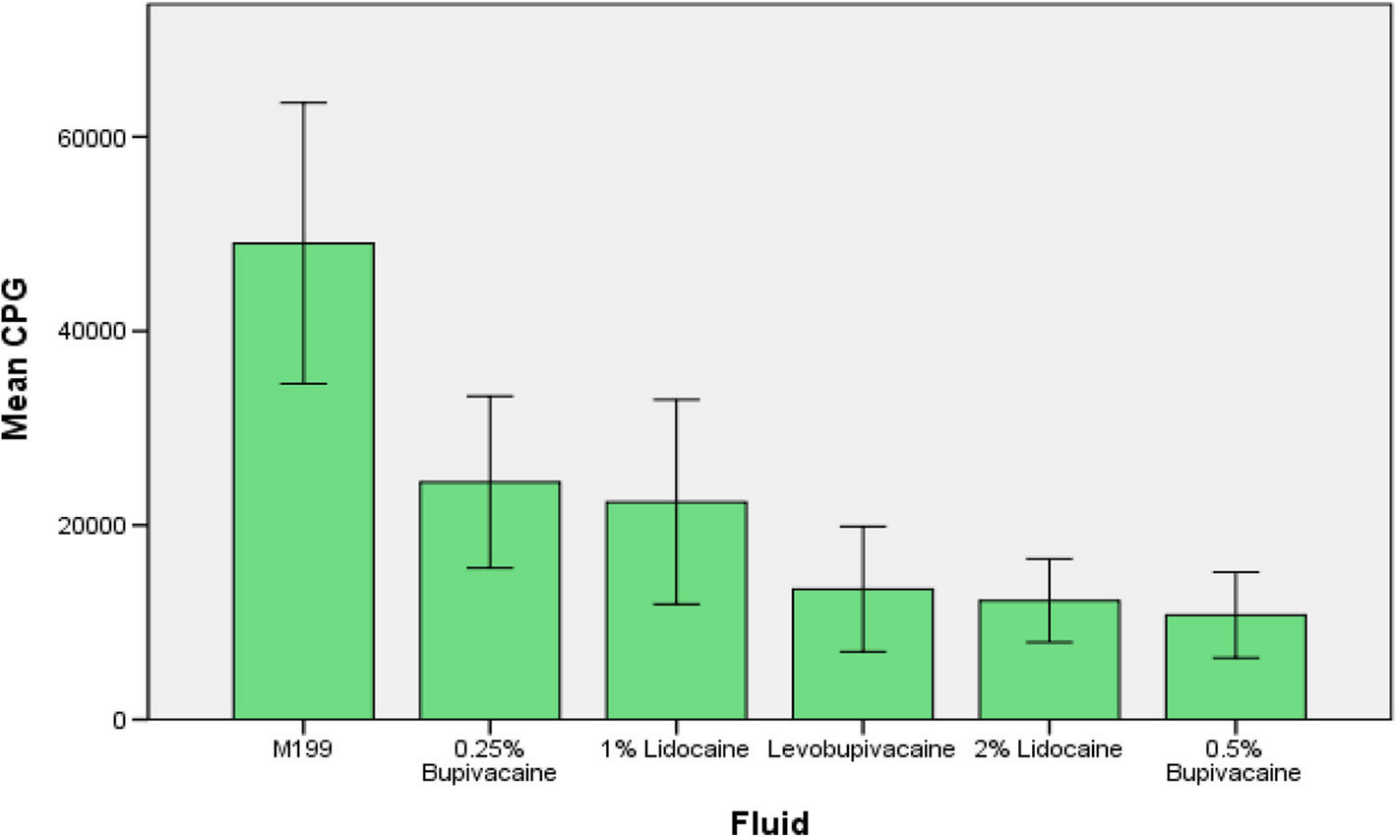
Percentage inhibition of proteoglycan metabolism compared to a control solution after exposure to different local anaesthetic solutions

Compared to the control M199 culture medium, all local anaesthetic solutions caused a reduction in uptake of ^35^SO_4_ (Fig. 2 and Table 2). The percentage inhibition of proteoglycan metabolism compared to M199 control was 65% for 1% Lidocaine (*p* < 0.001), 79% for 2% Lidocaine (*p* < 0.001), 61% for 0.25% Bupivacaine (*p* < 0.001), 85% for 0.5% Bupivacaine (*p* < 0.001) and 77% for 0.5% Levobupivacaine (*p* < 0.001).

**Table 2:** Uptake of 35SO4 by articular cartilage specimens exposed to different local anaesthetic solutions, glucosamine and control

Counts per gram per minute (CPG)				
Fluid	Mean	Std. deviation	95% Confidence Intervals	
1% Lidocaine	22,386	32,054	11,851	32,922
2% Lidocaine	12,215	13,101	7909	16,521
0.25% Bupivacaine	24,433	26,913	15,587	33,279
0.5% Bupivacaine	10,741	13,464	6315	15,166
0.5% Levo-bupivacaine	13,404	19,683	6934	19,874
M199	49,041	44,030	34,569	63,514
Gluc-protect	33,231	25,637	22,876	43,586
Gluc-repair	17,369	18,954	9713	25,025

Gluc-protect: Testing protective effect of Glucosamine

Gluc-repair: Testing reparative effect of Glucosamine

Higher concentrations caused more reduction of ^35^SO_4_ uptake; 2% Lidocaine being worse than 1% Lidocaine (*p* < 0.001) and 0.5% Bupivacaine worse than 0.25% Bupivacaine (*p* < 0.001). There was no significant difference between the ^35^SO_4_ uptake of 2% Lidocaine and 0.5% Bupivacaine or between 1% Lidocaine and 0.25% Bupivacaine.

The addition of Glucosamine at the same time as the local anaesthetic reduced the proteoglycan inhibition of 0.5% Bupivacaine from 85 to 43% (*p* < 0.001). Adding Glucosamine to the culture medium after one-hour exposure to the 0.5% Bupivacaine helped reduce the proteoglycan inhibition from 85 to 70% (*p* = 0.004).

## Discussion

This study found that all the local anaesthetic agents inhibited proteoglycan metabolism ranging from 61 to 85%: therefore our results support our hypothesis. An interest into the effect of local anaesthetics on articular cartilage was sparked by several reports of chondrolysis associated with the use of post-operative intra-articular pain pumps. While clinically this adverse effect has mainly been seen with continuous infusions via pain pumps, even a single exposure to local anaesthetics has been found to be potentially harmful in laboratory studies.

Chu et al. (2006) found that exposure to 0.5% Bupivacaine for 30 min caused 42% chondrocyte death in bovine articular cartilage. Reduced chondrocyte viability has since been found with different concentrations of Lidocaine, Bupivacaine and Ropivacaine in a dose and duration dependent manner (Lane and Brighton 1974; Mankin and Lippiello 1969). Our results show that all local anaesthetic solutions tested caused proteoglycan inhibition in articular cartilage. We also found that 2% Lidocaine and 0.5% Bupivacaine effects were worse than 1% Lidocaine and 0.25% Bupivacaine respectively. However, at equivalent clinical concentration, both Lidocaine and Bupivacaine were equally harmful.

Chu et al. (2010) conducted an in vivo study on rat cartilage and found reduced chondrocyte density 6 months after a single exposure to 0.5% Bupivacaine, indicating that this effect is maintained at least in the medium term. However, there are currently only four reported cases of chondrolysis associated with a single exposure to local anaesthetic (Bailie and Ellenbecker 2009) and these patients also underwent an arthroscopic procedure with the use of suture anchors and an irrigation fluid, the other suspected risk factors for chondrolysis. Scheffel et al. (2010) summarised 100 cases of PAGCL and noted that symptoms started between 42 and 730 days after surgery whilst a radiographic diagnosis was made between 90 and 1095 days post operatively.

Therefore, even with large doses of local anaesthetics administered via an infusion, it can take up to 3 years for the diagnosis to become apparent. With smaller doses, any adverse effects may take several years to develop. While a single injection of local anaesthetic may not lead to chondrolysis, it may stimulate a degenerative process. There may be damage leading to osteoarthritis many years later but this would be attributed to the pathology for which the original injection was administered or to the indication for arthroscopy, if the injection was given post operatively.

Repetitive injections may cause cumulative damage but this has not been studied yet in either in vivo or in vitro studies. The long term effects of local anaesthetic exposure can be tested via an in vivo study similar to that of Chu et al. (2006) with a single exposure to a local anaesthetic and longer recovery period 12 or more months.

Whilst the use of Glucosamine is clinically controversial (Towheed et al. 2005), very clear beneficial effects were observed in our laboratory-based study. The majority of clinical trials have examined the effects of oral Glucosamine but there is evidence that intra-articular injections are safe and can help improve symptoms of osteoarthritis (Crolle and D'Este 1980; Vajaradul 1981). We found that Glucosamine offered protection against the adverse effect of local anaesthetics to articular cartilage and reduced the inhibition of proteoglycan metabolism by 37%. It was also able to reverse this inhibition by 10%, 1 h after exposure to 0.5% Bupivacaine. While this reparative effect was not as dramatic as the protective effect, we only measured one recovery time period of 16 h. More time may have provided more benefit. Even after the addition of Glucosamine, 43% proteoglycan inhibition was observed. Whilst this may still appear alarming, it is however, similar to the 35% inhibition seen with a simple solution such as normal saline, which is regularly used clinically to irrigate joints during arthroscopic surgery.

The mechanism by which Glucosamine protected or repaired articular cartilage damaged by local anaesthetics is unknown. Such a protective effect could be due to a direct chemical interaction of Glucosamine with 0.5% Bupivacaine perhaps neutralising the anaesthetic effect of Bupivacaine. However this does not explain the marginal recovery of 35-S uptake when the reparative effect of Glucosamine was studied after the removal of 0.5% Bupivacaine. Therefore, it is probably more likely that this was due to a direct chondro-protective effect mediated via stimulation of proteoglycan synthesis.

With so much evidence emerging that local anaesthetics may not be safe for intra-articular injection, questions have been asked whether they are at all necessary for postoperative analgesia. Townshend et al. (2009) did not find any difference between visual analogue pain scores of patients who had Bupivacaine injection around the arthroscopic portals only and those who had an intra-articular injection. However, they assessed scores only at one time interval, one-hour after arthroscopy, and did not calculate the amount of oral opiate and non-opiate analgesia consumed in each group. Campo et al. (2012) injected patients’ knee joints with 10mls of saline or 0.5% Bupivacaine or 0.75% Ropivacaine after arthroscopy and found only a small improvement in analgesia offered by the addition of local anaesthetics. They felt that systemic analgesia should be preferred to local anaesthetic intra-articular injection in view of the several published reports of chondrotoxicity.

Anecdotally, the use of levo-bupivacaine (Chirocaine) seems to be increasing because of less cardiotoxicity compared to Bupivacaine. We did not find any difference in proteoglycan inhibition caused by levo-bupivacaine compared to Bupivacaine.

Some authors have attempted to investigate the mechanism of this local anaesthetic toxicity. Dragoo et al. (2010) suggested that this adverse effect could be due to the presence of epinephrine, the preservative sodium metabisulphite and the low pH of such solutions. Hennig et al. (2010) found that Bupivacaine with the preservative Methylparaben caused more chondrocyte death than Bupivacaine alone, 5 min after exposure but not at 30 min.

Chu et al. had previously demonstrated cell death due to Bupivacaine in their earlier studies (Chu et al. 2008; Chu et al. 2006) but did not find any difference in vivo in superficial cell viability or histological scores between preservative free Bupivacaine and saline control at any time interval from 1 week to 6 months (Chu et al. 2010). They did, however, find reduced chondrocyte density 6 months after exposure to Bupivacaine only indicating that preservatives are not the primary cause of toxicity.

Bogatch et al. (2010) did not find any adverse effects due to Bupivacaine with epinephrine or due to low pH of phosphate buffered saline (PBS) control. They found that there was a crystallisation reaction between the anaesthetic and the culture medium and with synovial fluid and wondered whether an incompatibility between the synovial fluid and the local anaesthetic was responsible for the chondrocyte toxicity. We wonder whether the damage was caused by the crystals or whether a third chondrotoxic chemical was formed as a result.

Grishko et al. (2010) found that local anaesthetics caused mitochondrial DNA damage in chondrocytes leading to cell death. While this may explain the mechanism of cell death at a molecular level, the effect of addition of epinephrine or preservatives or low pH on local anaesthetic toxicity is yet unclear and needs to be investigated further.

We acknowledge the limitations of an in vitro model and recognise that further clinical studies are required to confirm or refute our laboratory findings. Ideally this should be in the form of a randomised controlled trial with long term follow up comparing the effects of a single exposure or multiple injections of local anaesthetics with those who have no local anaesthetic exposure. The development of arthritis and requirement of total joint arthroplasty can be an outcome measure. However, this would be very difficult to conduct as patients may refuse to have local anaesthetic injections once presented with the published evidence regarding possible adverse effects. Also, it will be very difficult to match patients for all the risk factors for osteoarthritis. For this reason, a long term follow up study with cartilage uptake MRI sequences may be more realistic.

We do not know if the addition of Glucosamine will have any neutralising effect on the analgesic properties of the local anaesthetic solution. This will need to be further established with a clinical study.

We have used cartilage from elderly patients wherein the cellular changes due to ageing would have commenced. We also only investigated sulphate metabolism and not cell viability. We would have liked to use an assessment of cell viability to complement our study but the cost of confocal or fluorescent microscopy was well outside our study grant. The cartilage from the femoral heads would have exhibited the changes associated with ageing and may also have suffered further insult in the form of fracture haematoma and inflammation. However, the metabolic activity should still be comparable to that of young cartilage. Although we did not perform a power analysis our sample size of a minimum of 26 specimens was larger than most other published studies.

A possible method for obviating a proportion of the damage caused by intra-articular local anaesthetic would be to co-inject bupivacaine with glucosamine and to avoid other local anaesthetic agents altogether. Further work needs to be carried out to ascertain the merits of this technique.

## Conclusion

Our results showed that all local anaesthetic solutions inhibited proteoglycan metabolism in articular cartilage and the addition of Glucosamine was able to reduce the inhibition of metabolism caused by 0.5% Bupivacaine. Intra-articular injection of local anaesthetics requires careful consideration of risks and benefits. Further clinical studies are required to assess whether any clinically relevant damage occurs to a joint injected with local anaesthetic.

## References

[CR1] Anakwenze OA, Hosalkar H, Huffman GR (2010). Case reports: Two cases of glenohumeral chondrolysis after intraarticular pain pumps. Clin Orthop Rel Res.

[CR2] Anderson SL, Buchko JZ, Taillon MR, Ernst MA (2010). Chondrolysis of the Glenohumeral Joint After Infusion of Bupivacaine Through an Intra-articular Pain Pump Catheter: A Report of 18 Cases. Arthroscopy.

[CR3] Bailie DS, Ellenbecker TS (2009) Severe chondrolysis after shoulder arthroscopy: a case series. J Shoulder Elb Surg 18(5):742–747. 10.1016/j.jse.2008.10.017 Epub 2009 Jan 30 10.1016/j.jse.2008.10.017 19186080

[CR4] Bogatch MT, Ferachi DG, Kyle B, Popinchalk S, Howell MH, Ge D, You Z, Savoie FH. Is chemical incompatibility responsible for chondrocyte death induced by local anesthetics? Am J Sports Med 2010;38-3:520-526.PMID 20194957 10.1177/0363546509349799 20194957

[CR5] Bulstra SK, Kuijer R, Eerdmans P, Van der Linden AJ (1994). The effect in vitro of irrigating solutions on intact rat articular cartilage. J Bone Joint Surg.

[CR6] Campo MM, Kerkhoffs GM, In S, Weeseman RR, Van der Vis HM, Albers GH (2012). A randomised controlled trial for the effectiveness of intra-articular Ropivacaine and Bupivacaine on pain after knee arthroscopy: the DUPRA (Dutch Pain Relief afer Arthroscopy) trial. Knee Surg Sports Traumatol Arthros.

[CR7] Chan PS, Caron JP, Rosa GJM, Orth MW (2005). Glucosamine and chondroitin sulfate regulate gene expression and synthesis of nitric oxide and prostaglandin E2 in articular cartilage explants. Osteoarth Cart.

[CR8] Chu CR, Coyle CH, Chu CT, Szczodry M, Seshadri V, Karpie JC, Cieslak KM, Pringle EK (2010). In vivo effects of single intra-articular injection of 0.5% bupivacaine on articular cartilage. J Bone Joint Surg.

[CR9] Chu CR, Izzo NJ, Coyle CH, Papas NE, Logar A (2008). The in vitro effects of bupivacaine on articular chondrocytes. J Bone Joint Surg Br.

[CR10] Chu CR, Izzo NJ, Papas NE, Fu FH (2006). In Vitro Exposure to 0.5% Bupivacaine Is Cytotoxic to Bovine Articular Chondrocytes. Arthroscopy.

[CR11] Collins DH, McElligott TF (1960). Sulphate (35SO4) Uptake by Chondrocytes in Relation to Histological Changes in Osteo-Arthritic Human Articular Cartilage. Ann Rheum Dis.

[CR12] Crolle G, D'Este E (1980). Glucosamine Sulphate for the management of arthrosis: A controlled clinical investigation. Curr Med Res Opi.

[CR13] Dragoo JL, Korotkova T, Kim HJ, Jagadish A (2010). Chondrotoxity of low pH, epinephrine, and preservatives found in local anaesthetics containing epinephrine. Am J Sports Med.

[CR14] Fenton JI, Chlebek-Brown KA, Peters TL, Caron JP, Orth MW (2000). The effects of glucosamine derivatives on equine articular cartilage degradation in explant culture. Osteoarth Cart.

[CR15] Grishko V, Xu M, Wilson G (2010). Pearsall 4th AW. Apoptosis and mitochondrial dysfunction in human chondrocytes following exposure to lidocaine, bupivacaine, and ropivacaine. J Bone Joint Surg.

[CR16] Gulihar A, Bryson DJ, Taylor GJ (2013). Effects of different irrigation fluids on human articular cartilage: An in vitro study. Arthroscopy.

[CR17] Hansen BP, Beck CL, Beck EP, Townsley RW (2007). Postarthroscopic glenohumeral chondrolysis. Am J Sports Med.

[CR18] Hennig GS, Hosgood G, Bebenik-Angapen LJ, Lauer SK, Morgan TW (2010). Evaluation of chondrocyte death in canine osteochondral explants exposed to a 0.5% solution of bupivacaine. Am J Vet Res.

[CR19] Karpie JC, Chu CR (2007). Lidocaine exhibits dose- and time-dependent cytotoxic effects on bovine articular chondrocytes in vitro. Am J Sports Med.

[CR20] Lane JM, Brighton CT (1974). In vitro rabbit articular cartilage organ model. I. Morphology and glycosaminoglycan metabolism. Arthritis Rheum.

[CR21] Mankin HJ, Lippiello L (1969). The turnover of adult rabbit articular cartilage. J Bone Joint Surg Am.

[CR22] Meachim G, Collins DH (1962). Cell counts of normal osteoarthritic articular cartilage in relation to the uptake sulphate (35SO4) in vitro. Ann Rheum Dis.

[CR23] Oegema TR, Deloria LB, Sandy JD, Hart DA (2002). Effect of oral glucosamine on cartilage and meniscus in normal and chymopapain-injected knees of young rabbits. Arth Rheum.

[CR24] Parker BL (2006). Glucosamine and its effects on articular cartilage and osteoarthritis. Athl Ther Today.

[CR25] Piper SL, Kim HT (2008). Comparison of ropivacaine and bupivacaine toxicity in human articular chondrocytes. J Bone Joint Surg.

[CR26] Scheffel PT, Clinton J, Lynch JR, Warme WJ, Bertelsen AL, Matsen FA (2010). Glenohumeral chondrolysis: A systematic review of 100 cases from the English language literature. J Shoulder Elb Surg.

[CR27] Shikhman AR, Amiel D, D'Lima D, Hwang SB, Hu C, Xu A, Hashimoto S, Kobayashi K, Sasho T, Lotz MK (2005). Chondroprotective activity of N-acetylglucosamine in rabbits with experimental osteoarthritis. Ann Rheum Dis.

[CR28] Terry DE, Rees-Milton K, Pruss C, Hopwood J, Carran J, Anastassiades TP (2007). Modulation of articular chondrocyte proliferation and anionic glycoconjugate synthesis by glucosamine (GlcN), N-acetyl GlcN (GlcNAc) GlcN sulfate salt (GlcN.S) and covalent glucosamine sulfates (GlcN-SO4). Osteoarth Cart.

[CR29] Towheed TE, Maxwell L, Anastassiades TP, Shea B, Houpt J, Robinson V (2005). Glucosamine HYPERLINK "http://www.ncbi.nlm.nih.gov/pubmed/15846645" therapy for treating osteoarthritis. Cochrane Database Syst Rev.

[CR30] Townshend D, Emmerson K, Jones S, Partington P, Muller S (2009). Intra-articualr injection versus portal infiltration of 0.5% bupivacaine following arthroscopy of the knee: a rospective, randomised double blinded trial. J Bone Joint Surg Br.

[CR31] Vajaradul Y (1981). Double-blind clinical evaluation of intra-articular glucosamine in outpatients with gonarthrosis. Clin Therap.

